# Reproductive Performance and Culling Rate of Purebred Holstein Cows and Their Crosses With Fleckvieh and Brown Swiss Cows Under Subtropical Conditions

**DOI:** 10.3389/fvets.2021.752941

**Published:** 2021-11-16

**Authors:** Mohammed A. F. Nasr, Mohamed A. Hussein, Adel Q. Alkhedaide, Mahmoud S. El-Tarabany, ELshimaa M. Roushdy

**Affiliations:** ^1^Department of Animal Wealth Development, Faculty of Veterinary Medicine, Zagazig University, Zagazig, Egypt; ^2^Department of Clinical Laboratory Sciences, Turabah University College, Taif University, Taif, Saudi Arabia

**Keywords:** holstein cows, Brown Swiss cows, fleckvieh cows, reproduction, health

## Abstract

This study aimed to elucidate the reproductive performance of purebred Holstein (HO) cows with their crosses with Fleckvieh (FV) and Brown Swiss (BS) cows under subtropical conditions. A total of 677 cows [487 HO, 104 HO × FV (HFV); 50% FV and 50% HO and 86 HO × BS (HB); 50% BS and 50% HO] were enrolled in this study. Pure HO cows had significantly greater service per conception (S/C; 3.69), days open (147.9 days), and calving interval (449.6 days), than the HFV (2.89, 116.7, and 407.4 days, respectively) and HB (3.07, 134.3, and 434.2 days, respectively) crossbred cows. At day 28, the conception percentage was significantly greater among HFV crossbred cows vs. pure HO cows [crude odds ratios (COR) = 2.16], but embryonic loss, abortion percentage, calving difficulty, and retained placenta percentage were similar (*p* > 0.05) among pure HO cows and their crosses. HFV crossbreds had significantly lower incidence of endometritis (COR = 0.70, *p* = 0.035), mastitis (COR = 0.69, *p* = 0.015), and ketosis (COR = 0.53, *p* = 0.004) vs. other cows. HB and pure HO cows had a similar incidence of mastitis, lameness, and ketosis (COR = 0.76, 0.75, and 0.81; *p* = 0.223, 0.468, and 0.492, respectively). HFV crossbred cows had a lower risk of culling rate than HB crossbred cows. In summary, HFV cows demonstrated the best reproductive performance in terms of S/C, days open, calving interval, conception at 28 days, mastitis percentage, ketosis percentage, and endometritis.

## Introduction

Holstein (HO) cows are the most important dairy cattle breed around the world; therefore, emphasis should be given on improving their reproductive performance while maintaining high production (1). The genetic aspects of dairy cattle fertility can be manipulated (2–4), but these methods have been neglected in the selection programs. Consequently, days open (DO) and service per conception (S/C) has increased by 64 days and 1.1%, respectively (5). However, crossbreeding for a commercial dairy producer is difficult in terms of (a) identifying competitive dairy breeds for HO cows and (b) choosing the best method of crossbreeding. Regarding the methods of crossbreeding, terminal crossbreeding is allowing a maximum use of heterosis, whereas rotational crossbreeding is allowing the breeding of own replacements. On the other hand, rotational crossbreeding has been widely used to avoid the relatively high costs of the rearing period and the value of each individual animal (6, 7). In order for a program of HO crossbreeding to be effective, the reproductive performance must be better than that of purebred cows to economically offset the decreased milk production (8).

Reproductive efficiency is a fundamental tool for the profitability of seasonal-calving production systems and is fortified by the ability of cows to resume cyclicity early post-calving, express estrus, conceive, and maintain pregnancy (9). The reduced fertility of HO cattle is primarily due to the following factors: (a) alterations in the reproductive physiology of lactating cattle attributed to the physiological adaptations of high milk production (10–12); (b) an unfavorable genetic correlation among milk production parameter, fertility, and powerful selection for improved milk production (13); and (c) greater energy used by the mammary gland and postpartum uterine infection subsequently disrupting hormonal and metabolic status (14). The fertility of lactating cows has a crucial impact on the financial sustainability of a dairy farm. Reproductive failure triggers economic losses caused by increased calving intervals (CIs), elevated insemination expenses, reduced numbers of heifers for replacement, decreased income of bull calves marketing, and eventual culling of the lactating cows. In the UK, 44% of culling after the first season was attributed to the reproductive failure of the dairy cattle (15, 16).

Crossbreeding has been proposed as a fast method to reverse the decline of reproductive performance caused by “holsteinization” (17). Commercial dairy farmers have found that crossbred cows had superior conception rates vs. purebred HO cows (18). The promising benefits of crossbred cows over purebred cows include a quicker breeding phase and decreased DO with a greater percentage of conception (19–23). The period from parturition to first breeding was low for Normande × HO and Montbéliarde × HO cows, with a higher conception rate after the first service. Crossing may be improving the incidence of uterine disorders, as in case of the crossing Montbéliarde × HO cows compared to pure HO cows (24) as it will prolong the DO (25). The improvement in fertility could be attributed to the hybrid vigor of crossbreds, because the intensive selection in inbreeding may be reducing the fertility of pure HO cows (26). Moreover, F1 Holstein × Jersey cows had fewer DO (127 vs. 150 days) and a higher percentage of cows conceived by 150 days postpartum (75 vs. 59%) when compared with pure HO cows (27). Despite their high milk production efficiency, pure HO cows suffer from poor reproductive performance. In contrast, Fleckvieh (FV) and Brown Swiss (BS) cows are characterized by great reproductive performance with low milk production potential comparable to HO cows (28, 29).

FV cows are a Simmental-derived breed from Bavaria in Germany; it is a dual-purpose breed with moderate to high milk production with a superior fertility performance. FV sires with specialized dairy cows are preferable in a crossbreeding program, because they produce high-quality weaner calves for the feedlot without reducing the cow's milk production (30, 31). Fertility in dairy cows has declined due to intensive selection for milk production (14). HO cows are considered the highest milk-producing breed, but suffer from poor reproductive performance. In contrast, FV and BS cows are characterized by high reproductive performance, but with low milk production (28, 29). Consequently, the aim of this study is to explore the reproductive performance of purebred HO cows with their crosses of FV and BS cows under a subtropical climate.

## Materials and Methods

### Animals and Management

We investigated a herd of the Egyptian cattle at a breeding dairy farm (28 km Ismailia-Cairo desert road, Cairo, Egypt) that consisted of 677 cows (487 HO, 104 FV × HO, and 86 BS × HO). Animals were kept free in shaded open yards with a cool spraying system under hot conditions and divided based on their average daily milk production. Animals were fed *ad libitum* corn silage and total mixed ration (TMR) with citrus silage. The TMR was mixed daily and accustomed based on the amount of milk yield and body condition score of the cows. The TMR was formed to fulfill the ideal necessities of energy, protein, minerals, and vitamins, containing 24.83% neutral detergent fiber and 16.91% crude protein with 7.36 MJ/kg net energy for lactation. The feed through the dry period was prepared to fulfill the requirements commended by NRC. Water was freely available at all times. Heifers were artificially inseminated for the first time upon reaching a body weight of 350 kg. Estrus was detected using Afikim pedometers. Cows exhibiting estrus after 60 days postpartum were artificially inseminated. Insemination occurred approximately 8 h after a cow was first observed. Ultrasonography was used to detect the pregnancy at day 30 after service. Cows were machine milked thrice daily at 07:30, 16:30, and 23:30 in parallel parlor Afikim 32 points. The Afikim (4.1) computer program system was used in the farm. Cows were dried 2 months before the next expected calving.

### Reproductive Traits

Reproduction records of HO, HO × FV (F1 crossbreed; 50% FV and 50% HO), and HO × BS (F1 cows; 50% BS and 50% HO) cows were collected for the present study. The recorded reproductive traits were age at first calving (AFC), S/C, conception rate, pregnancy rate, embryonic loss rate, abortion rate, DO, and CI. AFC is the age of heifers at first parturition. S/C measures the herd fertility and is defined as the number of services required for the cow to be conceived. Conception rate (P/AI 30) was defined as the number of pregnant cows at day 30 post-insemination divided by the total number of cows inseminated. Pregnancy rate (P/AI 75) was defined as the number of pregnant cows at day 75 post-insemination divided by the total number of cows inseminated. Embryonic loss rate was assessed as the number of cows confirmed non-pregnant at 75 days post-insemination divided by the total number of cows recognized pregnant at 30 days post-insemination. Abortion rate was assessed as the number of aborted cows from 75 to 210 days of gestation divided by the total number of cows recognized pregnant at day 75 post-insemination. DO, or the calving-to-conception interval, was defined as the number of days between cows calving and conception, and Calving interval (CI) was defined as the time interval between two successive calving.

### Health Traits

Health traits were obtained from the herd records and usually diagnosed and confirmed by the herd veterinarian. Calving difficulty was defined as the need for assistance, to major force or surgery being required to extract the newborn. Retained placenta was defined as failure to expel fetal membranes within 24 h after parturition. Clinically, endometritis was defined as a cervix ≥7.5 cm in diameter by transrectal palpation at day 20 or more after parturition or as the presence of mucopurulent or purulent vaginal discharge by vaginoscopy after day 26 of parturition. The presence of clinical mastitis was detected by milkers upon observing a hard-swollen warm udder and changes in milk consistency (watery or blood-tinged secretions and clots in milk). Lameness was diagnosed as an infectious interdigital disease that included digital dermatitis (hairy heel warts) and heel horn erosion and foot rot (interdigital phlegmon and interdigital necrobacillosis).

### Statistical Analysis

Data were gathered and analyzed by the General Linear Model procedures of the IBM SPSS software program (Version 16.0; IBM Corp., NY, USA). Regarding the reproductive traits (age at first calving, service per conception, days open, and calving interval), the normality of distribution was confirmed by Kolmogorov–Smirnov test. Also, the interquartile range (IQR) was applied to exclude any extreme outliers from data set. The statistical model was explained as follows:


$${\text{Y}}_{\text{ijk}}=\mu {+\text{G}}_{\text{i}}{+\text{A}}_{\text{j}}+\;{\text{e}}_{\text{ijk}}$$

where Y_ijk_ is an observation of each trait, μ is the overall mean, G_i_ is the fixed effect of the genetic type i (1, 2, and 3), A_j_ is the effect of the age at first calving, and e_ijk_ is the random effect assumed to be distributed with mean zero and variance σ2e.

The univariate logistic regression model was adopted through the maximum likelihood process to estimate the effect of genetic type (categorized into three levels) on conception rate, pregnancy, embryonic loss, abortion rates, and health traits (calving difficulty, retained placenta, endometritis, mastitis, lameness, and ketosis). The HO cows were used as the reference category for the comparison of crude odds ratios (CORs). Results are presented as percentages with the assessed odds ratios and the 95% confidence interval (95% CI). Statistical significance was set at *p* < 0.05. Cox's proportional hazard model (PHREG) was used to evaluate the consequence of predictor variables (genetic type and season of calving) on embryonic loss. The time variable used in the model was days from calving to first AI or censored. The predictor variables tested were genetic type (three levels), and season of calving (winter, spring, summer, and autumn) as categorical variables. The final model only includes one predictor variable (genetic type). The outcomes are presented as hazard ratios.

## Results

### Reproductive Traits of Purebred HO and Crossbred Cows (HFV and HB)

Pure HO cows had significantly greater S/C vs. HFV crossbreds by 21.68%. Furthermore, they had significantly greater DO (147.9 days) and CI (449.6 days) than HFV (Table 1). Moreover, the description for reproductive indices and health traits of purebred Holstein, Holstein × Fleckvieh, and their Holstein × Brown Swiss cows is presented in Table 2.

**Table 1:** Age at first calving, service per conception rate, days open and calving interval of purebred Holstein, Holstein × Fleckvieh, and Holstein × Brown Swiss cows.

**Trait**	**Genetic type**				
	**HO^1^**	**HFV^2^**	**HB^3^**	**SEM^4^**	***p*** **-value**
Age at 1st calving (month)	24.6	24.8	24.2	0.18	0.399
S/C^5^	3.69^a^	2.89^b^	3.07^ab^	0.14	0.003
Days open (day)	147.9^a^	116.7^b^	134.3^ab^	3.76	0.001
Calving interval (day)	449.6^a^	407.4^b^	434.2^ab^	5.29	0.001

1 *HO, purebred Holstein*;

2 *HFV, F_1_crossbred Holstein × Fleckvieh (50% HO, 50% FV)*;

3 *HB, F_1_crossbred Holstein × Brown Swiss (50% HO, 50% BS)*;

4 *SEM, standard error of means*;

5 *S/C, service per conception*;

a, b, ab *Means with different superscripts in each row were significantly different*.

**Table 2:** Description for reproductive indices and health traits of purebred Holstein, Holstein × Fleckvieh, and their Holstein × Brown Swiss cows.

**Indices (%)**	**Genetic type**		
	**HO^1^**	**HFV^2^**	**HB^3^**
Number	487	104	86
Conception (P AI 28 days)	21.8	37.6	28.7
Embryonic loss	10.4	7.8	8.3
Abortion	13.4	12.4	11.3
Calving difficulty	47.9	42.8	46.9
Retained placenta	19.5	20.4	16.9
Endometritis	11.8	8.6	9.5
Mastitis	32.3	24.8	26.5
Lameness	19.2	16.8	15.1
Ketosis	35.1	22.3	30.2

1 *HO: purebred Holstein*;

2 *HFV, F_1_crossbred Holstein × Fleckvieh (50 % HO, 50 % FV)*;

3 *HB, F_1_crossbred Holstein × Brown Swiss (50 % HO, 50 % BS)*.

### Reproductive Indices of Purebred HO and Crossbred Cows

Conception percentage (P/AI at day 28) was significantly greater in HFV crossbred cows vs. pure HO cows (COR = 2.16), whereas this was not significantly different between pure HO cows and HB crossbred cows. Embryonic loss and abortion percentage were similar (*p* > 0.05) between pure HO cows and their crosses (Table 3).

**Table 3:** Odds ratio for reproductive indices of purebred Holstein, Holstein × Fleckvieh, and Holstein × Brown Swiss cows.

**Indices**	**Genetic type**		
	**HO^1^**	**HFV^2^**	**HB^3^**
**Conception (P AI 28 days)**			
^4^OR	R	2.16	1.53
95% CI	-	1.41–3.29	0.79–2.97
^5^SE	-	0.22	0.33
*p-*value	-	0.003	0.204
**Embryonic loss**			
^4^OR	R	0.72	0.75
95% CI	-	0.36–1.44	0.25–2.20
^5^SE	-	0.35	0.39
*p-*value	-	0.360	0.595
**Abortion**			
^4^OR	R	0.92	0.83
95% CI	-	0.53–1.61	0.34–2.04
^5^SE	-	0.28	0.36
*p-*value	-	0.773	0.683

1 *HO, purebred Holstein*;

2 *HFV, F_1_crossbred Holstein × Fleckvieh (50% HO, 50% FV)*;

3 *HB, F_1_crossbred Holstein × Brown Swiss (50% HO, 50% BS)*;

4 *OR, odds ratio (95% confidence interval); R: reference value (HO) and*

5 *SE, standard error*.

### Reproductive Health Indices of Purebred HO and Crossbred Cows

Calving difficulty and retained placenta percentage were similar (*p* > 0.05) between pure HO cows and their crosses (Table 3). HFV crossbreds had a significantly low incidence of endometritis percentage vs. other cows (COR = 0.70, *p* = 0.035). However, the incidence rates of calving difficulty (COR = 0.95, *p* = 0.872), retained placenta (COR = 0.84, *p* = 0.381), and endometritis percentage (COR = 0.78, *p* = 0.309) of HB crossbred cows were comparable to those of pure HO cows (Table 4).

**Table 4:** Odds ratio for reproductive problems of purebred Holstein, Holstein × Fleckvieh, and Holstein × Brown Swiss cows.

**Indices**	**Genetic type**		
	**HO^1^**	**HFV^2^**	**HB^3^**
**Calving difficulty**			
^4^OR	R	0.81	0.95
95% CI	-	0.55–1.17	0.52–1.73
^5^SE	-	0.19	0.30
*p-*value	-	0.262	0.872
**Retained placenta**			
^4^OR	R	1.06	0.84
95% CI	-	0.84–1.34	0.57–1.23
^5^SE	-	0.12	0.19
*p-*value	-	0.611	0.381
**Endometritis**			
^4^OR	R	0.70	0.78
95% CI	-	0.51–0.97	0.48–1.26
^5^SE	-	0.17	0.24
*p-*value	-	0.035	0.309

1 *HO, purebred Holstein*;

2 *HFV, F_1_crossbred Holstein × Fleckvieh (50% HO, 50% FV)*;

3 *HB, F_1_crossbred Holstein × Brown Swiss (50% HO, 50% BS)*;

4 *OR, odds ratio (95% confidence interval); R, reference value (HO) and*

5 *SE, standard error*.

### General Health Indices of Purebred HO and Crossbred Cows

Lameness percentage was similar (*p* > 0.05) between pure HO cows and their crosses. HFV cows had a general health aptitude that was confirmed with a low prevalence of mastitis (COR = 0.69, *p* = 0.015) and ketosis percentage (COR = 0.53, *p* = 0.004). However, the incidence rates of mastitis, lameness, and ketosis in HB crossbred cattle (COR = 0.76, 0.75, and 0.81; *p* = 0.223, 0.468, and 0.492, respectively) were similar to those of pure HO cows (Table 5).

**Table 5:** Odds ratio for general health indices of purebred Holstein, Holstein × Fleckvieh, and Holstein × Brown Swiss cows.

**Indices**	**Genetic type**		
	**HO^1^**	**HFV^2^**	**HB^3^**
**Mastitis**			
^4^OR	R	0.69	0.76
95% CI	-	0.51–0.93	0.47–1.19
^5^SE	-	0.15	0.22
*p-*value	-	0.015	0.223
**Lameness**			
^4^OR	R	0.84	0.75
95% CI	-	0.52–1.38	0.34–1.65
^5^SE	-	0.25	0.29
*p-*value	-	0.498	0.468
**Ketosis**			
^4^OR	R	0.53	0.81
95% CI	-	0.34–0.82	0.43–1.50
^5^SE	-	0.22	0.30
*p-*value	-	0.004	0.492

1 *HO, purebred Holstein*;

2 *HFV, F_1_crossbred Holstein × Fleckvieh (50% HO, 50% FV)*;

3 *HB, F_1_crossbred Holstein × Brown Swiss (50% HO, 50% BS)*;

4 *OR, odds ratio (95% confidence interval); R, reference value (HO) and*

5 *SE, standard error*.

### Estimates of Cox Proportional Hazard Ratio for the Culling Rate of Primiparous Purebred HO and Crossbred Cows (Time Variable Was the Age at Calving)

**Figure 1:**
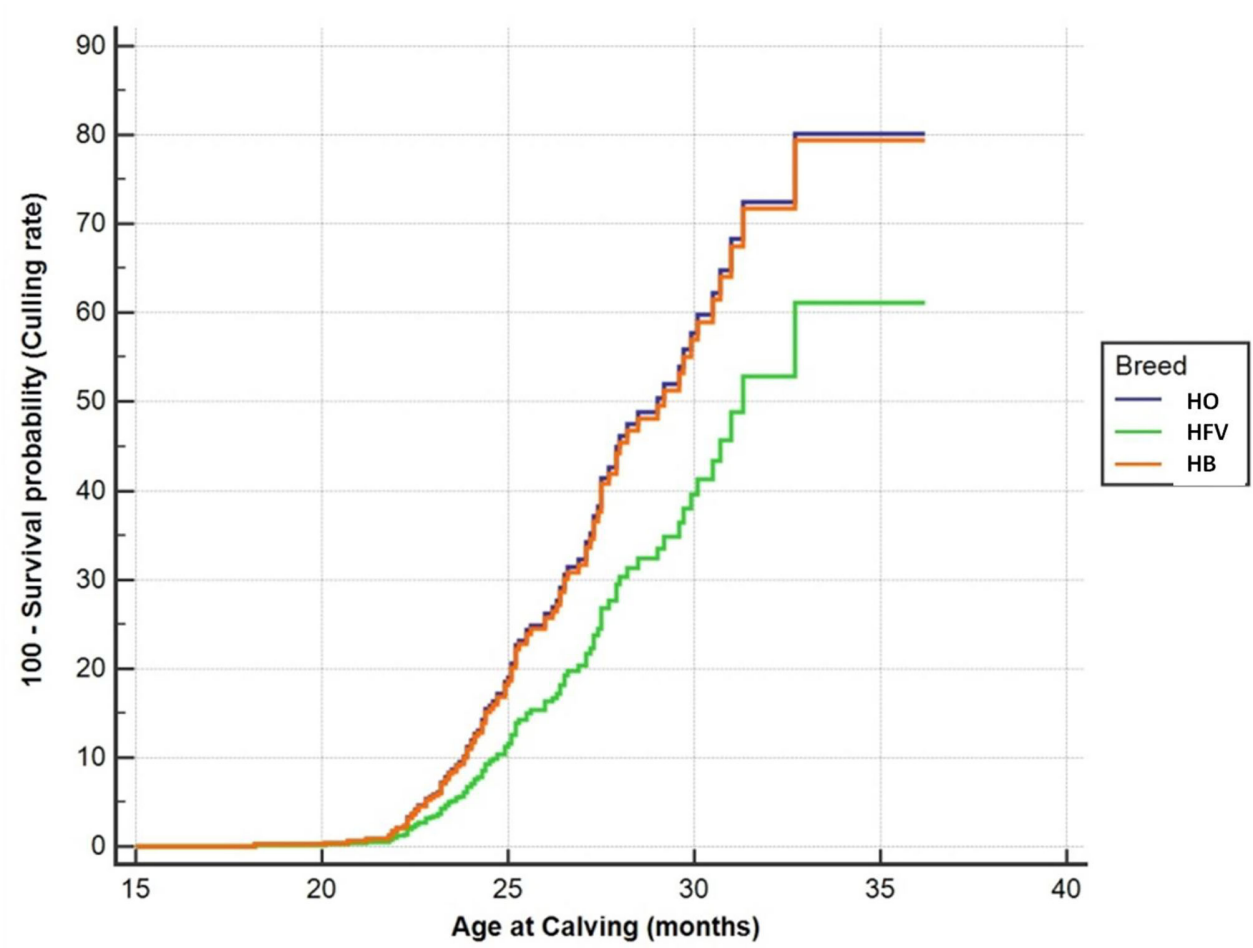
Hazard function for culling rate in primiparous purebred Holsteins (HO) and their crossbred cows. HFV, Holsteins × Fleckvieh; HB, Holstein × Brown Swiss. The time variable was the age at calving.

The Cox regression model demonstrated that HB crossbred cows had no significant association with the hazard of culling rate, while this was significantly with HFV crossbred cows. Accordingly, HFV crossbred cows had a higher risk of culling rate than HB crossbred cows (hazard ratio = 0.58 and 0.97, *p* = 0.026 and 0.892, respectively) (Table 6, Figure 1).

**Table 6:** Hazard ratio for the culling rate in primiparous purebred Holsteins and their crossbred cows (time variable was the age at calving).

**Factor**	***b***	**SE**	**Hazard ratio**	**95% CI**	***p*** **-value**
**Genetic type**					
HO^1^	-	-	R		-
HFV^2^	−0.538	0.24	0.58	0.37–0.93	0.026
HB^3^	−0.022	0.31	0.97	0.51–1.88	0.892

1 *HO, purebred Holstein*.

2 *HFV, F_1_crossbred Holstein × Fleckvieh (50% HO, 50% FV)*.

3 *HB, F_1_crossbred Holstein × Brown Swiss (50% HO, 50% BS). B, coefficient of regression; R, reference value (HO) and SE: standard error*.

## Discussion

This research aimed to assess the reproductive performance of purebred HO cows and their crosses under a subtropical climate condition. In our study, pure HO cows had significantly greater S/C, DO, and CI compared to their crosses (HFV and HB cows). Previous studies have determined that crossbred cattle had lower DO vs. purebred HO cows (127 vs. 150 days) (32, 33). Normande × HO, Montbéliarde × HO, and Scandinavian Red × HO cows also had lower DO versus pure HO cows (122, 124, 131, and 147 days, respectively) (32). These results support our findings.

Crossbred Holstein × Simmental cows had higher conception rate, shorter CI, and shorter calving to first service interval vs. pure HO cows when conventional semen was used (34). The same is true for crossing pure HO and FV cows (35). The S/C for crossbred cows was fewer than that of HO cows during the first lactation (36, 37). The present study revealed that HFV crossbred cows had significantly fewer days from calving to conception (DO), S/C, and CI than pure HO cows, similar to previous studies. HFV cows cycled earlier and exhibited estrus quickly. HO × Jersey cows have been reported to cycle earlier after parturition when compared with purebred HO cows (38). Similarly, Swalve et al. (33) reported that BS × HO crossbred cows had less DO compared to pure HO cows. These results may be attributed to the extended anovulatory periods in pure HO cows (39), a late resumption of luteal activity (39–42), environmental influences and management properties associated with the reproduction (mainly estrus detection), and the existence of infections in reproductive organs that can harmfully upset fertility (33).

Various investigations have reported that HO cows require more days to be conceived compared to BS and crossbred cows, which could be attributed to their susceptibility to thermal stress (43). Meanwhile, Knob et al. (34) did not detect any difference of age at first calving between HO and their crossbreed with Simmental. In contrast, the present study clearly showed that crossbred cows (HO × FV) had superior reproductive performance than pure HO. These findings may display the heterosis and complementarity between these breeds (44).

In the present study, conception percentage (P/AI at day 28) in HFV crossbred cows was significantly superior vs. pure HO cows, but this was not significantly different between pure HO and HB crossbred cows; these findings are consistent with those recently reported in purebred HO cows (45). A larger percentage of crossbred cows were conceived at 150 and 180 days postpartum when compared with purebred cows (75 vs. 59% and 77 vs. 61%, respectively) (27). Moreover, a higher percentage of HO × FV conceived within 100 days postpartum with fewer days from calving to first service (48% and 86 days, respectively) compared to pure HO cows (37% and 104 days, respectively) (35). Moreover, the percentage of pregnant cows at 100 days postpartum was higher for FV × HO cows vs. pure HO cows. These results may be because the pure HO cows took longer to resume ovarian activity and cycling postpartum vs. Jersey × HO and Jersey cows (38).

Embryonic loss and abortion percentage were similar between pure HO cows and their crosses. However, a recent study recorded a significantly lower embryonic loss rate in BS × HO cows compared to pure HO cows (29). The results of the current investigation suggest a general enhancement of the reproductive performance and general health attributes (lower incidence of endometritis, mastitis, and ketosis) of HFV crossbreeds than of pure HO. The incidence of calving difficulty, retained placenta, and lameness were similar between pure HO cows and their crosses. These results were analogous to a recent study on HO and BS cows and their crossbreeds (29). Several studies have determined that diseases associated with the reproductive tract are correlated to these findings and that the DO and CI could also be impacted in addition to the general reproductive efficacy (46).

New trials have stated that the frequency of metritis was higher in pure HO cows than in crossbreeds (47). However, the current outcomes showed a conflict with the previous studies concerning HO × BS backcrosses (8), which found that health traits were similar between pure HO cows and their backcrosses. This implies that backcrossed animals did not improve the animal's health traits. Calving difficulty and retained placenta percentage were similar among pure HO cows and their crosses. Pure HO cows, compared to BS cows, had a higher prevalence of health disorders during their lactations (41 vs. 14%) and mastitis (19 vs. 3%) (28), which supported our findings. Lameness was also similar between pure HO cows and their crosses. Our results were similar to that of a recent study on pure HO cows and their backcrosses (48).

The culling judgments for individual cows were affected by production, fertility, age, health, lactation phase, cull value of cows, value of replacements, or a combination of these factors (46). In the present study, the Cox regression model revealed a significant relationship between cow breed and the hazard of culling. The HFV crossbred cows had a lower culling rate than HB crossbred cows. The higher longevity of HFV crossbred cows may be associated with their superior fertility, because this is considered the foremost reason of culling in dairy farms (36). Consistent with these findings, Knob et al. (34) reported a higher survival rate for HO × Simmental cows, especially on the second parturition. Moreover, there was a significant and relatively large increase (+13.4%) of the survival to the second calving of Scandinavian Red × HO crosses compared with purebred HO cows (49). The lower survival rate of pure HO cows may be attributed to their higher calving difficulty compared to crossbred cows. Recently, Clasen et al. (50) found a much greater survival rate from the first to third calving (+15%) for Nordic Red × HO crossbreds vs. purebred HO cows. In contrast, Hazel et al. (36) did not find a significant difference in survival rate from the first and second calving between Viking Red × HO crossbreds vs. purebred HO cows.

## Conclusion

Reproductive efficiency is a fundamental tool for the profitability of seasonal-calving production systems and is fortified by the ability of cows to resume cyclicity early post-calving, express estrus, conceive, and maintain pregnancy. Milk production and fertility had an unfavorable genetic association with forceful selection for augmented milk production. Therefore, crossbreeding has been proposed as a fast method to reverse the decline of reproductive performance. HFV cows demonstrated the best reproductive performance (in terms of S/C, DO, CI, conception at 28 days, mastitis, ketosis, and endometritis) with a lower risk of culling rate because HFV cows resume cyclicity earlier after calving vs. HO cows, even in similar milk production situations.

## Data Availability Statement

The raw data supporting the conclusions of this article will be made available by the authors, without undue reservation.

## Ethics Statement

This work was performed in accordance with the guidelines of the Animal Care and Use Committee of the Zagazig University (this study only involved retrospective records).

## Author Contributions

MN, AA, ME-T, and ER: conceptualization, data curation, formal analysis, investigation, methodology, resources, validation, visualization, roles/writing—original draft, writing—review, and editing. MH: conceptualization, data curation, formal analysis, investigation, methodology, validation, visualization, roles/writing—original draft, writing—review, and editing. All authors contributed to the article and approved the submitted version.

## Funding

Taif University Researchers supporting Project number (TURSP-2020/104) Taif University, Taif, Saudi Arabia.

## Conflict of Interest

The authors declare that the research was conducted in the absence of any commercial or financial relationships that could be construed as a potential conflict of interest.

## Publisher's Note

All claims expressed in this article are solely those of the authors and do not necessarily represent those of their affiliated organizations, or those of the publisher, the editors and the reviewers. Any product that may be evaluated in this article, or claim that may be made by its manufacturer, is not guaranteed or endorsed by the publisher.
